# Cancer stem cells: a target for overcoming therapeutic resistance and relapse

**DOI:** 10.20892/j.issn.2095-3941.2023.0333

**Published:** 2024-02-05

**Authors:** Shuo Zhang, Rui Yang, Yujie Ouyang, Yang Shen, Lanlin Hu, Chuan Xu

**Affiliations:** 1 Department of Radiation Oncology, Radiation Oncology Key Laboratory of Sichuan Province, Sichuan Clinical Research Center for Cancer, Sichuan Cancer Hospital & Institute, Sichuan Cancer Center, Affiliated Cancer Hospital of University of Electronic Science and Technology of China, Chengdu 610042, China; 2 Department of Ultrasound in Medicine, Chengdu Wenjiang District People’s Hospital, Chengdu 611130, China; 3 Acupuncture and Massage College, Chengdu University of Traditional Chinese Medicine, Chengdu 611137, China; 4 Department of Oncology & Cancer Institute, Sichuan Academy of Medical Sciences and Sichuan Provincial People’s Hospital, University of Electronic Science and Technology of China, Chengdu 610072, China; 5 Sichuan Provincial Key Laboratory for Human Disease Gene Study, Department of Laboratory Medicine, Sichuan Academy of Medical Sciences and Sichuan Provincial People’s Hospital, University of Electronic Science and Technology of China, Chengdu 610072, China; 6 School of Pharmacy, Macau University of Science and Technology, Macau SAR 999078, China; 7 Yu-Yue Pathology Scientific Research Center, Chongqing 400039, China; 8 Jinfeng Laboratory, Chongqing 401329, China

**Keywords:** Cancer stem cells, therapeutic resistance, metabolism, immunology, biomarkers

## Abstract

Cancer stem cells (CSCs) are a small subset of cells in cancers that are thought to initiate tumorous transformation and promote metastasis, recurrence, and resistance to treatment. Growing evidence has revealed the existence of CSCs in various types of cancers and suggested that CSCs differentiate into diverse lineage cells that contribute to tumor progression. We may be able to overcome the limitations of cancer treatment with a comprehensive understanding of the biological features and mechanisms underlying therapeutic resistance in CSCs. This review provides an overview of the properties, biomarkers, and mechanisms of resistance shown by CSCs. Recent findings on metabolic features, especially fatty acid metabolism and ferroptosis in CSCs, are highlighted, along with promising targeting strategies. Targeting CSCs is a potential treatment plan to conquer cancer and prevent resistance and relapse in cancer treatment.

## Introduction

Cancer stem cells (CSCs) are a specific subpopulation of tumor cells with stem cell-like capacities of self-renewal and differentiation that were originally proposed to exist by Mackillop in 1983^1^. The CSC theory hypothesizes that tumor initiation, metastasis, and recurrence are favored by a small number of CSCs present in tumors^2^. Over the years studies have identified CSCs in various types of cancers, including leukemia^3^, breast cancer^4^, colorectal cancer (CRC)^5^, and lung cancer^6^. CSCs are mostly, but not necessarily found in a mitotically dormant or quiescent state. CSCs can potentially differentiate into different lineage cells, such as cancer cells, vascular endothelial cells, pericytes, and erythroblasts^7–10^. Recent studies have also revealed the phenotypic and functional heterogeneity of CSCs during tumor progression^11^. Therefore, targeting CSCs could be an effective therapeutic approach to eradicate the source of cancer cells and combat therapeutic resistance, ultimately transforming the therapeutic paradigm for cancers and improving patient prognosis.

This review discusses recent advances pertaining to CSCs, including biological features, biomarkers, and mechanisms underlying resistance to different therapies. We have focused on the metabolic and immunologic aspects of CSCs and the potential therapeutic implications of targeting CSCs to overcome resistance to cancer treatment.

## Biological properties of CSCs

Several studies have investigated the characteristics of CSCs in distinct types of cancers, as thoroughly reviewed elsewhere^12–14^. In this section we will focus on recent discoveries on the immunologic and metabolic properties of CSCs.

### Immunologic properties of CSCs

**Figure 1:**
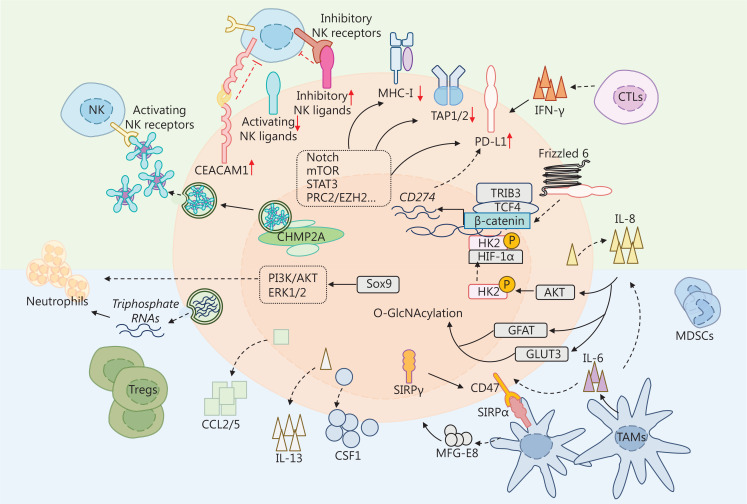
Immunologic properties of cancer stem cells (CSCs). CSCs escape immune surveillance by altering immunomodulatory molecules. CSCs avoid recognition by immune cells by downregulating MHC class I (MHC-I) molecules and antigen processing machinery (APM) molecule [antigen processing 1/2 (TAP1/2)]. CSCs evade NK-mediated killing by decreasing the expression of and shedding activating NK ligands (ULBP1-6, MICA/B, Nkp30L, and Nkp44L) and increasing inhibitory NK ligands (KIR2DL1-5 and KIR3D1-3), and carcinoembryonic antigen-related cell adhesion molecule 1 (CEACAM1). High levels of MICA/B are secreted through membrane vesicles facilitated by increased levels of charged multivesicular body protein (CHMP2A) in CSCs. CSCs also express high levels of immune checkpoints, such as programmed death-ligand 1 (PD-L1), which interact with corresponding receptors on immune cells and thereby impede the activation and proliferation of immune cells. The expression and stability of PD-L1 is regulated by multiple signaling pathways, including Notch, mTOR, phosphoinositide 3-kinase (PI3K)/AKT, signal transducer, and activator of transcription-3 (STAT3), as well as interleukin and interferon-γ (IFN-γ). IFN-γ is released by CSCs or other immune cells. Interleukin-8 (IL-8) can be secreted by CSCs and tumor-associated macrophages (TAMs). IL-8 induces phosphorylation and nuclear translocation of hexokinase 2 (HK2) by AKT. Phosphorylated HK2 and hypoxia-inducible factor-1 alpha (HIF-1α) promote the transcription of CD274 by binding the promoter. PD-L1 also binds to Frizzled 6 to activate β-catenin signaling and further upregulates PD-L1. IL-8 enhances O-GlcNAcylation by upregulating glucose uptake transporter 3 (GLUT3) and glutamine fructose-6-phosphate aminotransferase (GFAT), which contribute to stemness in CSCs. Moreover, CSCs secrete cytokines, chemokines, and triphosphate RNAs to recruit immunosuppressive immune cells. Sox9/C-C motif chemokine ligand 1 (CCL1) axis recruits neutrophils through PI3K/AT and ERK1/2 signaling. CSCs release triphosphate RNAs to recruit neutrophils. Additionally, CSCs secrete CCL2/5, IL-13, and CSF1 to recruit inhibitory Tregs and TAMs. TAMs release milk-fat globule-epidermal growth factor-VIII (MFG-E) and IL-6 to maintain the stemness of CSCs. Moreover, the increasing CD47 in CSCs interacts with signal regulatory protein alpha (SIRPα) to exert a “don’t eat me” signal. SIRγ is highly expressed in CSCs to provoke a “don’t eat me” signal.

A previous study showed that epigenetic immunoediting may drive an acquired immune evasion program in the most aggressive mesenchymal glioblastoma multiforme (GBM) subtype by modifying the tumor immune microenvironment^15,16^. Recent research indicates heterogeneous immunomodulatory molecules in CSCs and crosstalk between CSCs and stromal cells in the tumor microenvironment (TME)^17^. This section summarizes the immunomodulatory molecules found in CSCs, with a particular focus on major histocompatibility complex (MHC) molecules, natural killer (NK) ligands, and immune checkpoints (**Figure 1**). The interplay between CSCs and immune cells, as well as other stromal cells, which have been extensively reviewed in other sources, are also briefly discussed^18–21^.

#### Surface immunoregulatory molecules in CSCs

CD8^+^ cytotoxic T cells have a vital role in eliminating tumors by recognizing and killing tumor cells that display foreign antigens presented by MHC-I molecules. Several studies have shown that dysregulation of antigen presentation-related molecules in CSCs contributes to immune evasion. In various types of cancers, such as melanoma, lung cancer, GBM, and head and neck squamous cell carcinoma (HNSCC), CSCs exhibit a reduction or deficiency of MHC-I/II molecules *via* an *in vitro* tumorsphere formation assay^22–25^. The CSC-enriched tumorspheres from murine TC-1 lung cancer cells have lower expression of surface MHC-I molecules than other tumorspheres, which makes the tumorspheres resistant to human papillomavirus (HPV) 16 E6/E7 peptide vaccine-mediated killing. In a tumorsphere-bearing mouse model, less CD8^+^ CTL infiltration is found in CSC-enriched tumorspheres^23,25^. Similarly, in HNSCC cell lines, CD44^+^ CSCs exhibit low levels of HLA-A2, HLA-II, and antigen processing 2 (TAP2) expression, making it difficult for cytotoxic T cells (CTLs) or NK cells to identify the CD44^+^ CSCs^25,26^. An analysis of tumor-associated antigens (TAAs) and the antigen processing and presentation molecule (APM) in tumorspheres from 12 human solid tumor cell lines indicated that weak or deficient expression of HLA-I/II molecules was detected in 9 cell lines, whereas the increasing expression of APMs, such as low molecular mass protein-2/7 (LMP2/7), multi-catalytic endopeptidase complex subunit 1 (MECL-1), and TAP1/2, was observed in 12 cell lines^26^. Further studies in immunocompetent and immunocompromised mice have demonstrated that aldehyde dehydrogenase (ALDH)^+^, but not CD44^+^CD24^−^ breast CSCs, have TAP1 genes and the co-stimulatory molecule, CD80, that are downregulated by DNA hypermethylation, which causes impairment in T cell-mediated killing^27^. Lastly, a genome-wide CRISPR/Cas9 screen has revealed that polycomb repressive complex 2 (PRC2) epigenetically downregulates the MHC-I in an EZH2-dependent manner, prompting resistance to T cell-mediated killing^28^.

Generally, cells with low expression or absence of MHC-I molecules are susceptible to attack by NK cells, which suggests the potential for NK cell-mediated CSC killing^29–31^. However, CSCs in GBM exhibit resistance to lysis mediated by resting NK cells due to MHC class I molecule expression, as reported by Avril et al.^32^ Activation of NK cells by lectins restores GSC sensitivity to NK lysis. Additionally, upregulated expression of NKG2DL augments NK-mediated killing in glioma CSCs and drug-resistant ovarian cancer cells^33,34^. CSCs isolated from CRC patients express lower levels of MHC-I molecules and higher levels of the activating NK ligands, Nkp30L and Nkp44L, making the CSCs isolated from CRC patients more susceptible to NK cell-mediated killing^35^. According to a report by Luna et al.^36^, the proteasome inhibitor, bortezomib, induces the expression of CSC-related genes and the activation of NKG2DL MHC class I chain-related molecules A/B (MICA/B) in ALDH^+^ and ALDH^−^ cells from GBM, synovial sarcoma, and pancreatic adenocarcinoma cell lines. Bortezomib sensitizes ALDH^+^ cells to NK cell-mediated killing in *in vitro* and *in vivo* models^36^. A mechanistic study involving reprogrammed CSCs from liver cancer revealed that CD44 mRNA functions as a competing endogenous RNA (ceRNA) that specifically binds microRNA (miR)-34a, thereby preventing the activating NKG2DL, UL16 binding protein 2 (ULBP2), from degradation. Stable expression of ULBP2 facilitates NK cells to kill liver CSCs^37^; however, several studies have indicated that CSCs evade the innate immune response by increasing inhibitory NK ligands, which decreases the expression of activating NK ligands or shedding activating NK ligands^38–41^. Tumors remove natural killer group 2D (NKG2D) ligands, which is one of the primary mechanisms responsible for subverting NKG2D-mediated immunosurveillance in leukemia stem cells (LSCs)^39^. Similarly, MICA/B downregulation is modulated by oncogenic miR-20a and leads to the resistance of CSCs to NK cell cytotoxicity, as well as lung cancer metastasis, in breast CSCs^42^. Furthermore, CD24^−/low^/CD44^+^ CSCs isolated from radiotherapy-resistant triple-negative breast cancer (TNBC) cells exhibit reduced MICA/B expression and profound expression of the inhibitory NKG2A ligand, HLA-E. In an *in vivo* mouse model, CD24^−/low^/CD44^+^ CSCs recruit NK cells to the peritumoral area but deprive CD24^−/low^/CD44^+^ CSCs of cytotoxicity^43^. Additionally, EpCAM^high^ hepatocellular carcinoma (HCC) cells that express high levels of carcinoembryonic antigen-related cell adhesion molecule 1 (CEACAM1) display CSC properties and are not susceptible to NK killing^44^. Upregulated CEACAM1 is also detected in tumorspheres with CSC properties developed from liver cancer cells. The tumorspheres express low levels of ULBP1 and MICA/B on the cell surface, whereas elevated levels of soluble MICA are detected in conditioned medium from tumorspheres, which impedes NK cell-mediated killing^40^. The whole genome CRISPR–Cas9 screening system has identified the vital role of chromatin-modifying protein/charged multivesicular body protein (CHMP2A) in desensitizing CSCs to NK-mediated killing in HNSCC and GBM cells. Specifically, CHMP2A induces CSCs to secrete extracellular vesicles (EVs) expressing MICA/B and tumor necrosis factor (TNF)-related apoptosis-inducing ligand (TRAIL), thereby inducing apoptosis in NK cells^41^.

High levels of immune checkpoint molecules have been detected in CSCs, which hamper the immune response^45^. PD-L1 expression is higher in CSCs from various types of cancer, including breast cancer, squamous cell carcinoma, endometrial cancer, CRC, and non-small cell lung cancer (NSCLC)^45–49^. Numerous studies have identified different mechanisms underlying PD-L1 regulation in cancer, which have been thoroughly reviewed^50^. Hsu et al.^51^ reported that inhibition of PD-L1 expression with etoposide leads to an increase in tumor-infiltrating T cells. PD-L1 is modulated by diverse signaling pathways in CSCs, such as Wnt, phosphoinositide 3-kinase (PI3K)/AKT, Notch, mTOR, signal transducer and activator of transcription-3 (STAT3), and epigenetic signaling^46,51–56^. Notch 3 induces PD-L1 expression through mTOR and maintains stemness in PD-L1^high^ CSCs from breast cancer^46^. Sun et al.^52^ reported that interleukin-8 (IL-8) derived from gastric mesenchymal stem cells promotes PD-L1 expression *via* the STAT3/mTOR/c-Myc axis in CSCs from gastric cancer. IL-8 derived from gastric mesenchymal stem cells, along with AKT, promotes the phosphorylation and nuclear localization of HK2, which binds to HIF-1α and facilitates PD-L1 transcription^56^. Studies in CSCs from CRC organoids have suggested that tribble pseudokinase (TRIB3) recruits transcription factor 4 (TCF4) and β-catenin to the promoters of Wnt target genes, which in turn induces TRIB3 expression and maintains their stemness^57^. Administration of selective Wnt inhibitors or activators leads to a reduction and increase in PD-L1 expression in ALDH^+^/CD44^+^ CSCs from TNBCs, respectively, indicating the positive regulation of PD-L1 by Wnt^53^. The Wnt/β-catenin and PI3K/AKT pathways cooperate to promote tumorigenesis and resistance to therapy in CSCs from leukemia. β-catenin binds to loci on multiple immune checkpoint genes, including PD-L1, T-cell immunoglobulin domain mucin domain 3 (TIM3), and CD24. Targeting AKT inhibits this process, leading to a decrease in PD-L1, TIM3, and CD24 expression^55^. Moreover, PD-L1 promotes the activation of β-catenin and β-catenin CSC-associated target genes *via* an interaction with the receptor, Frizzled 6^48^. The expression and stability of PD-L1 is regulated in an epigenetic and posttranslational manner, respectively^51,54,58,59^; however, the expression landscape of immunomodulatory molecules varies between cancer types and individuals. The underlying regulatory mechanisms are also sophisticated and heterogeneous. Further understanding of molecular mechanisms can contribute to an improvement in CSC-targeted immunotherapy efficacy.

#### Crosstalk between CSCs and the TME

The interplay between CSCs and immune cells in the TME has a crucial role in the evasion of immune surveillance, which enables the survival and growth of CSCs. CD44^+^ CD90^+^ CSCs have a higher tendency for lymph node metastasis in small-cell lung cancer (SCLC), which promotes the response of cytotoxic T lymphocytes (CTLs). The expression of PD-L1, which is promoted by the secretion of IFN-γ by activated CTLs, leads to adaptive resistance capacity, resulting in prolonged inflammation and upregulation of regulatory ligands that ultimately impair the proliferative capacity and cytotoxicity of CTLs^60^. Research has shown that regulatory T cells (Tregs) promote cancer stemness in gliomas through the TGF-β/NF-κB/IL-6/STAT3 signaling axis. The anti-IL-6 receptor antibody, tocilizumab, shows efficacy in inhibiting tumor growth and stemness induced by Tregs in glioma xenograft models^60,61^ Tregs are subpopulations of CD4^+^ T cells that suppress the immune response by inhibiting the activation of NK cells and the cytotoxic function of CD8^+^ T cells^62^. Other subpopulations of CD4^+^ T cells include Th1, Th2, and Th17 cells. Th1 cells secrete IL-2 and interferons, which activate the proliferation of CD8^+^ T cells and NK cells. Th2 cells promote the maturation and clonal proliferation of B cells by secreting cytokines, such as IL-4 and IL-6^62^. CSCs secrete the chemokines, C-C motif chemokine ligand 1 (CCL1), CCL2, and CCL5, which recruit Tregs^19^. The balance of Th17/Treg cells is closely related to tumor immunity and has a critical role in tumor progression^63^. Cytokines secreted by CSCs, including CCL5, MKN-45, IL-6, and IL-8, have been shown to affect the Th17/Treg balance, which has been reviewed elsewhere^64^.

In the context of cancer, M2 macrophages are commonly regarded as tumor-associated macrophages (TAMs)^65,66^. Activation of Yes-associated protein (YAP) in hepatocellular CSCs leads to tumorigenesis and TAM recruitment^67^. CSCs secrete chemokines, such as CCL2, CCL5, colony stimulation factor 1 (CSF1), growth differentiation factor 15 (GDF15), IL-13, and transforming growth factor-β (TGFβ), as well as periostin and Wnt-induced signaling protein 1 [WISP1 (also known as CCN4)], which may impact the polarization state of TAMs and promote tumorigenesis^19^. TAMs secrete IL-6, which promotes HCC carcinogenesis by stimulating CSC-like characteristics. Tocilizumab disrupts TAM-enhanced CSC expansion in HCC^68^. The cytokine, IL-8, which is produced by various cells, including macrophages and monocytes, acts as a chemotactic cytokine to bring neutrophils to inflammatory or injured sites^69^. IL-8 has been shown to enhance O-GlcNAcylation, but not glycolysis mediated by the upregulation of glucose uptake transporter 3 (GLUT3) and glutamine fructose-6-phosphate aminotransferase (GFAT), which promotes the generation and maintenance of CSCs in colon and lung cancer cells. The effect of O-GlcNAcylation on CSCs has not been fully elucidated^70^. TAMs have been shown to secrete CCL5, which mediates the self-renewal of prostatic CSCs and metastases^71^. Jinushi and colleagues^72^ reported that CD44^+^/ALDH^+^ colon CSCs and CD133^+^/ALDH1^+^ lung CSCs induce secretion of the milk-fat globule-epidermal growth factor-VIII (MFG-E8) by TAMs, which with IL-6 maintains the activity and promotes the therapeutic resistance of CSCs. Additionally, the “don’t eat me” signal, CD47, is upregulated in CSCs from several cancer types. CD47 binds to myeloid-specific signal regulatory protein alpha (SIRPα) on macrophages, impeding phagocytosis and allowing immune evasion^73–75^. Our previous study identified a population of cancer cells expressing SIRPγ. SIRPγ^hi^ cancer cells display stemness-related properties and contribute to immune escape signals by sustaining CD47 expression, which halts macrophage-mediated phagocytosis in SIRPγ^hi^ and SIRPγ^lo/−^ tumor cells^6^.

Several studies have explored the immunosuppressive role of neutrophils in cancer progression. One study by the Szczerba group^76^ identified an interaction between neutrophils and circulating cancer cells that contributes to metastasis in patients and mouse models of breast cancer. Circulating cancer cells have stem cell characteristics and are precursors of metastasis^77^. Sox9 has been identified as a CSC marker in HCC. The Sox9/CXCL5 axis activates PI3K/AKT and ERK1/2 signaling, promoting the proliferation and invasion of HCC cells, as well as infiltration of intratumoral Ly6G^+^ neutrophils in the F4/80+ macrophage orthotopic xenograft model^78^. Hwang et al.^79^ discovered that exosomes released by colorectal CSCs prolong the lifespan of neutrophils by activating PRR/NF-κB signaling through exosomal triphosphate RNAs, leading to the expression of IL-1β and accelerating tumorigenesis.

Myeloid-derived suppressor cells (MDSCs) are a heterogeneous lineage of immature myeloid cells that can be divided into two major subsets [polymorphonuclear (PMN)-MDSCs and monocytic (M)-MDSCs]. MDSCs enhance ovarian cancer stemness by upregulating miR-101 and downregulating C-terminal binding proteins (CtBP2)^80^. Shidal et al.^81^ showed that miR-92a enhances integrin and TGF-β expression in CD133^+^ melanoma CSCs and leads to increased immunosuppressive cell phenotypes, including granulocytic MDSCs (gMDSCs) and Tregs. Another study found that ALDH1A1 promotes MDSC expansion by stimulating the secretion of GM-CSF, which is activated by the TGF-β-activated kinase 1 (TAK1)/NF-κB signaling pathway^82^.

Dendritic cells (DCs) also have a role in CSC maintenance. The interaction between C-X-C motif chemokine receptor 4 (CXCR4) expressed by follicular lymphoma (FL) cells with CSC-like activities and CXCL12, which is secreted by follicular DCs, facilitates chemotherapy resistance and tumorigenicity^83^. Natural killer T (NKT) cells are a specialized subtype of T cells that can be categorized into two types of cells [invariant NKT cells (iNKT cells) and type II NKT cells]. Although the role of NKT cells in CSCs has not been reported, iNKT cells release a variety of proinflammatory and anti-inflammatory cytokines that affect DCs, macrophages, neutrophils, NK cells, and T cells, which exert effects on CSCs^84^. B lymphocytes have a critical role in promoting and inhibiting tumor development. For example, B lymphocytes secrete cytokines, such as IL-10, TGF-β, and IL-35, and exhibit inhibitory effects by interacting with tumor tissues and lymphocytes, such as T cells, APCs, Tregs, and MDSCs^85^.

Cancer-associated fibroblasts (CAFs) and adipocytes also affect the stemness of CSCs. Studies conducted *in vitro* have shown that CAFs promote the expression of stem cell markers [CD44, SRY-box 2 (Sox2), and Bmi-1], as well as the self-renewal and expansion of CSCs by secreting cytokines, growth factors, androgen receptor-regulated factors, and exosomes^86–90^. CAFs also induce the tumorsphere-forming phenotype in breast cancer cells by producing CCL2, which activates the Notch signaling pathway^91^. CAFs boost breast CSC proliferation by secreting stromal-derived-factor-1 (SDF-1), which activates the Wnt/β-catenin and PI3K/AKT signaling pathways^92^. Adipocytes maintain the stemness of CSCs by secreting more resistin, which upregulates stemness-related transcription factors [Octamer-binding transcription factor 4 (Oct4), Sox2, Nanog homeobox (Nanog), and ALDH1] and activates stemness-related pathways (Notch and Wnt/β-catenin)^93–96^. Adipocytes also shield breast CSCs treated with doxorubicin by secreting more resistin, which mediates the activation of the AMPK/mTOR and JNK pathways^97^.

The TME is an intricate and dynamic system comprising tumor cells, immune cells, fibroblasts, extracellular matrix, and other interconnected components. The interaction between CSCs and infiltrated immune cells is particularly important. However, the specific mechanism of immune evasion in CSCs and the interplay with the TME, especially NKT cells, B lymphomas, and neutrophils, have not been extensively explored and warrant further investigation.

### Metabolic properties of CSCs

Tumors are highly adaptive to metabolic perturbations^98^. Under hypoxic conditions, mitochondrial oxidative phosphorylation (OXPHO) is replaced by glycolysis to compensate for deficient mitochondrial machinery^99^. During nutrient deficiency, autophagy is one of the essential strategies for preserving cell viability^100–102^. Mounting evidence has revealed the unique metabolic features of CSCs, such as aberrant glucose consumption, excessive lactate production, and inefficient ATP production^103,104^. Depending on the availability of oxygen and nutrients, as well as other stromal cells in the TME, CSCs show heterogeneity in different tissues^105^. Glioma CSCs exhibit high metabolic plasticity because glioma CSCs switch metabolism to glycolytic metabolism when OXPHOS is blocked^106^. Studies involving different tumors, including osteosarcoma, GBM, breast cancer, lung cancer, ovarian cancer, nasopharyngeal carcinoma (NPC), HCC, and colon cancer, suggest that CSCs have higher glycolytic potential and less mitochondrial oxidative metabolism than other differentiated tumor cells^103,107–113^. The mitochondrial circRNA for translocating phosphoglycerate kinase 1 (mcPGK1) is in high levels in liver CSCs. mcPGK1 has a crucial role in regulating cell metabolism by inhibiting OXPHOS and promoting glycolysis, which changes the levels of specific chemicals, such as α-ketoglutaric acid and lactic acid. These changes, in turn, activate the Wnt/β-catenin pathway and promote self-renewal of liver CSCs. Additionally, mcPGK1 helps introduce PGK1 to the mitochondria by interacting with TOM40 and reprograms cell metabolism from oxidative phosphorylation to glycolysis *via* the PGK1-PDK1-PDH axis^114^. Gu et al.^115^ reported that the absence of miR-192-5p increases glycolysis by upregulating glucose transporter type 1 (GLUT1), 6-phosphofructo-2-kinase/fructose-2,6-biphosphatase3 (PFKFB3), and c-Myc, which inhibit the transcription of miR-192-5p and maintain high glycolytic activity in HCC cells. High glycolytic activity in HCC cells produces excess lactic acid, which activates ERK phosphorylation in co-cultured LX2 and THP1 *via* the N-Myc downstream regulatory gene 3 (NDRG3) and monocarboxylate transporters 1 (MCT1), and promotes tumor stemness^115^. Another study showed that HectH9 is an activator of glucose metabolism. HectH9 does this by mediating the K63-linked ubiquitin of hexokinase 2 (HK2), which then regulates the location of HK2 in mitochondria. Regulation of HK2 in mitochondria is essential in inducing glycolysis and preventing apoptosis. Conversely, blocking the HectH9/HK2 pathway leads to an increase in reactive oxygen species (ROS), which inhibits CSC expansion and the development of tumors^116^. Although controversial, some investigations have shown that CSCs rely more on mitochondrial oxidative metabolism^117–123^. Pancreatic CSCs derived from patient-derived xenograft (PDX) models have a preference for mitochondrial metabolism to survive^124^. Similarly, ovarian CSCs highly express genes related to mitochondrial OXPHOS and fatty acid oxidation^121^. In breast cancer, increasing mitochondrial bulk in CSCs maintains stem-like characteristics, metastatic potential, and resistance to DNA damage^125^. Moreover, some subpopulations of cells from various tumors, such as CD133^+^ CSCs in GBM and pancreatic ductal adenocarcinoma, ROS^low^ quiescent cells in leukemia, and side population cells in lung and breast cancer, have been profiled to express the OXPHOS phenotype^118,119,122,126^. Notably, both OXPHOS and glycolysis are active in ovarian CSCs^121,127^.

**Figure 2:**
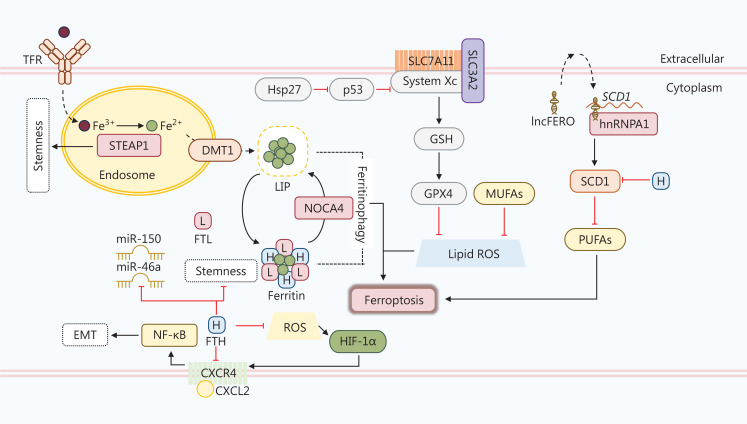
Ferroptosis in CSCs. The role of ferroptosis in CSCs is controversial. First, enzymes and transporters in the ferroptosis pathway are altered in some CSCs, including sterol regulatory-element binding protein 1 (STEAP1), divalent metal transporter protein 1 (DMT1), and nuclear receptor coactivator (NOCA4). The ferritin heavy chain (FTH) is downregulated in CSCs. The absence of FTH induces the expression of stemness-related genes, microRNA-150 (miR-150), and miR-46a, which maintain the stemness of CSCs. FTH also inhibits the activation of CXCR4/CXCL2 signaling and subsequent NF-κB-mediated EMT directly or by blocking ROS-regulated HIF-1α. A high level of SLC7A11, which is promoted by heat shock protein 27 (Hsp27)-mediated inhibition of p53, protects CSCs from ferroptosis *via* the GSH/GPX4 axis. Moreover, lnc-ENDOG-1:1 (lncFERO) secreted by CSCs interacts with stearoyl-CoA-desaturase (SCD1) mRNA and recruits hnRNPA1 to facilitate the translation of SCD1. SCD1 inhibits PUFA synthesis and thereby curbs ferroptosis. SCD1 is also inhibited by FTH in CSCs.

Several studies have revealed that fatty acid metabolism, especially the mevalonate pathway, is fundamental to the maintenance of stemness in CSCs^121,128–130^. Fatty acid oxidation (FAO) helps overcome glucose starvation and contributes to chemotherapeutic resistance in epithelial ovarian CSCs^131^. Consumption of the dietary fat palmitic acid has also been linked to increased metastatic potential in CSCs of oral squamous cell carcinoma (OSCC)^132^. Sterol regulatory-element binding protein 1 (SREBP-1) regulates genes involved in lipid metabolism, such as acetyl-CoA carboxylase (ACC) and fatty acid synthase (FASN). According to a study in cisplatin-resistant NSCLC cells, SREBP-1/SCAP/FASN signaling lowers CSC sensitivity to cisplatin. Treatment with fatostatin, an SREBP inhibitor, reverses cisplatin resistance and hampers stemness^133^. Dysregulation of lipid metabolism is associated with the maintenance of CSC stemness and poor survival. Further research should seek to clarify the function of SREBP-1/SCAP/FASN signaling in cisplatin resistance. Polyunsaturated fatty acids (PUFAs) and monounsaturated fatty acids (MUFAs) are the primary lipid metabolites and key regulators of ferroptosis, a form of cell death triggered by disturbances in metabolic networks, such as iron metabolism, mitochondrial metabolism, and lipid metabolism, and characterized by the accumulation of markers of lipid peroxidation^134^. Ferroptosis can be triggered by drugs, ionizing radiation, and cytokines, thereby suppressing tumor growth. However, ferroptosis can also encourage tumor growth by promoting inflammation-associated immunosuppression and other signaling pathways^135^. Recent research has highlighted the critical role and targeting potential of ferroptosis in CSCs (**Figure 2**). For example, a study reported that ferroptosis inducers selectively kill a mesenchymal breast cancer subpopulation with CSC properties through a non-apoptotic mechanism of action mediated by ROS in an iron-dependent manner^136^. Furthermore, Turcu et al.^137^ reported that the natural compound, salinomycin, selectively kills CD44^high^/CD24^low^ CSCs from breast cancer by interacting with lysosomal iron, which promotes ROS production and causes lysosomal membrane permeabilization. Intracellular ferric iron is primarily bound to transferrin (TF) and is imported *via* transferrin receptor 1 (TFR1). CSCs from breast and ovarian carcinomas express higher levels of TFRs, which induce iron uptake and sensitize CSCs to agents inducing ferroptosis^138,139^. The six-transmembrane epithelial antigen of prostate 3 (STEAP3) is a ferrireductase located at the plasma membrane that catalyzes the reduction of Fe^3+^ to Fe^2+^ after lysosome-endosome fusion. Fe^2+^ is then released from the endosome into a labile iron pool (LIP) in the cytoplasm, which is regulated by divalent metal transporter protein 1 (DMT1) and contributes to iron homeostasis. Elevated STEAP3 promotes the proliferation and stemness of CSCs in gliomas^140^. Inhibition of DMT1 also causes accumulation of lysosomal iron and ROS, leading to ferroptosis^137^; however, a study involving non-CSC glioblastoma cells demonstrated that temozolomide induces ferroptosis by upregulating DMT1 expression and increasing iron content^141^. Ferritin is composed of a heavy chain (FTH) and a light chain (FTL), and can store > 4,000 iron atoms and convert Fe^2+^ to Fe^3+^. Ferritinophagy is the process by which nuclear receptor coactivator 4 (NCOA4) releases the iron stored in ferritin to LIP and triggers ferroptosis^142^. A study conducted on osteosarcoma CSCs showed that exposure to a static magnetic field stimulates NCOA4-mediated ferritinophagy, promoting CSC proliferation and self-renewal^143^. Higher levels of glutathione peroxidase 4 (GPX4) and cystine/glutamate antiporter solute carrier family 7 member 11 (SLC7A11) protect esophageal CSCs from ferroptosis, which is induced by increased intracellular iron content. *In vitro* experiments have shown that heat shock protein 27 (Hsp27) positively regulates SLC7A11/GPX4 by downregulating p53^144^. Epithelial-to-mesenchymal transition (EMT) leads to the acquisition of CSC properties. FTH-mediated ROS dysregulation promotes C-X-C motif ligand 12 (CXCL12)/C-X-C motif chemokine receptor 4 (CXCR4) axis activation and EMT in erythroleukemia K562 cells^145^. Studies have also reported that FTH expression modulates EMT in *in vitro* models of breast and lung cancer^146,147^. The results of another study suggested that FTH silencing leads to an imbalance in the metabolism of unsaturated fatty acids and overexpression of stem cell markers in ovarian cancer^148^. Furthermore, gastric cancer (GC)-secreted exosomal lnc-ENDOG-1:1 (lncFERO) promotes stearoyl-CoA-desaturase (SCD1) translation by recruiting heterogeneous nuclear ribonucleoprotein A1 (hnRNPA1), then inhibits ferroptosis and enhances stemness in gastric CSCs *in vitro* and *in vivo*^149^. The roles of fatty acid metabolism and ferroptosis in CSCs from various types of cancer are clearly controversial.

In addition to glucose and fatty acid metabolism, other metabolic signaling pathways, such as glutamine metabolism and lysine catabolism, are enhanced in CSCs. CSCs rely heavily on glutamine in pancreatic cancer, so limiting the availability of CSCs can reduce the limited self-renewal of CSCs and enhance the sensitivity of CSCs to radiation therapy, followed by an increased level of ROS in cell lines and mouse models^150^. Elevated lysine catabolism has been detected in colon adenocarcinoma circulating tumor cells, which exhibit a high capacity for colonization in the liver and an active Wnt signaling pathway^151^. Despite the findings mentioned above, the full extent of metabolic networks in CSCs is not fully understood. In recent decades, altered metabolism in cancer and non-cancer cells in the TME has been considered a potential target for cancer treatment and relapse prevention. In this regard, a thorough understanding of CSC metabolism is essential for developing effective treatments and preventing cancer relapse.

## Biomarkers of CSCs

Because CSCs have a vital role in tumorigenesis and therapeutic resistance, it is essential to identify this specific population in cancer tissues. Numerous studies have investigated molecular biomarkers for CSCs in cancer cells, mouse models, and patient tissues. In addition to the classic biomarkers, such as CD133, CD44, epithelial cell adhesion molecule (EpCAM), and CD90, other biomarkers have also been studied in specific cancers. In this section, we will summarize the current knowledge about CSC biomarkers in different types of cancers, including cell-surface molecules, transcription factors, and other CSC markers (**Table 1**).

**Table 1:** CSC biomarkers in different cancer types

Biomarkers	Cancer types
CD133	Lung cancer^152–154^, colon cancer^155,156^, prostate cancer^157^, ovarian cancer^158^, melanoma^159^, osteosarcoma^160,161^, leukemia^162^, hepatocellular carcinoma (HCC)^163^, pancreatic cancer^164^, and oral squamous cell carcinoma (OSCC)^165^
CD44	Colorectal cancer (CRC)^166–168^, pancreatic cancer^169^, ovarian cancer^170^, gastric cancer^171,172^, prostate cancer^157,173^, non-small cell lung cancer (NSCLC)^174^, OSCC^175^, nasopharyngeal carcinoma (NPC)^176^, HCC^168,177,178^
CD90	Murine breast cancer^179^, HCC^180^, gastric cancer^181^, esophageal squamous cell carcinoma (ESCC)^182^, lung cancer^183^, pancreatic cancer^184^, glioma^185,186^, insulinoma^187^, HCC^188^
EpCAM	Breast cancer^189,190^, CRC^166^, HCC^188,191^, NPC^176^, pancreatic cancer^192,193^
Lgr5	Gastric cancer^194,195^, pancreatic cancer^196,197^, HCC^198,199^, CRC^200,201^, ovarian cancer^202^, cervical cancer^203^, breast cancer^204^
Oct4	Glioma^205^, pancreatic cancer^206^, HCC^207^, breast cancer^208^, prostate cancer^209,210^, bladder cancer^211^, ovarian cancer^212^, lung cancer^213^
Sox2	Head and neck squamous cell carcinoma (HNSCC)^214^, medulloblastoma^215^, glioma^216^, breast cancer^217^, gastric cancer^218^, CRC^219^, lung cancer^220^, cervical cancer^221^, melanoma^222^, osteosarcoma^223^, ovarian cancer^224^, pancreatic cancer^225^, bladder cancer^226^, skin cancer^227^
Klf4	Leukemia^228^, anaplastic meningioma^229^, CRC^230^, gastric cancer^231^, NSCLC^232^, HCC^233^, bladder cancer^234^, ESCC^235^
Nanog	Glioblastoma (GBM)^236^, leukemia^237^, lung cancer^238^, breast cancer^239^, ESCC^240^, gastric cancer^241^, CRC^242^, ovarian cancer^243^, prostate cancer^244^, HCC^245^, HNSCC^246^, renal cancer^247^
c-Myc	Neuroblastoma^248^, lung cancer^249^, CRC^250^, breast cancer^251^
ALDH	Ovarian cancer^252,253^, lung cancer^254–256^, breast cancer^257,258^, cervical cancer^259^, HCC^260^, colon cancer^261,262^
Bmi-1	Gastric cancer^263^, HNSCC^5,264^, CRC^265^, ESCC^266–268^, hematopoietic neoplasm^269^, glioma cancer^270,271^
Nestin	Neurogenic tumors^272–276^, rhabdomyosarcoma^277^, osteosarcoma^278^, chondrosarcoma^160^, fibrosarcoma^160^, ovarian^279–281^, OSCC^282^, prostate cancer^283,284^, gallbladder cancer^285^, lung cancer^286^, colon cancer^287^, breast cancer^288,289^, gastric cancer^290^, pancreatic cancer^291^
Msi1	CRC^292^, OSCC^293,294^, neuronal cancer^295^
Tim-3	Small-cell lung cancer (SCLC)^60^, acute myelogenous leukemia (AML)^296^
CXCR4	Glioma^297^, prostate cancer^298^

### Cell surface molecules

Cell surface molecules are practical for isolating CSCs by flow cytometry or magnetic sorting, as well as specific targeting. Many surface markers have been identified in CSCs, such as CD133, CD44, CD90, EpCAM, leucine-rich repeat-containing G protein-coupled receptor 5 (Lgr5), CD13, CD19, CD20, CD24, CD26, CD27, and CD34. Among the surface markers, CD133, CD44, CD90, EpCAM, and Lgr5 are the most investigated markers in CSCs.

#### CD133

CD133 is a transmembrane glycoprotein initially characterized as a hematopoietic stem cell and neuroepithelial stem cell marker^299,300^. In 2004 Singh et al.^273^ isolated a subpopulation of cells expressing CD133, possessing self-renewal potential, and the ability to recapitulate the original tumor from brain tumors. *In situ* injection of 100 CD133^+^ tumor cells in non-obese, diabetic, severe combined immunodeficient (NOD-SCID) mouse brains successfully produced a tumor. CD133 is a CSC biomarker of various cancer types, including lung cancer^152–154^, colon cancer^155,156,177^, prostate cancer^157^, ovarian cancer^158^, melanoma^159^, osteosarcoma^160,161,278^, leukemia^162^, HCC^163^, pancreatic cancer^164^, and OSCC^165^. CD133 alone might not be sufficient to identify CSCs, so other biomarkers are required. A few studies have also reported that CD133^+^ cells fail to recapitulate the original tumor and that CD133^−^ cells have the potential to produce tumors in mouse models^167,301–303^.

#### CD44

CD44 is a transmembrane glycoprotein that was first used as a CSC marker in breast cancer^189^. In 9 of 10 breast cancer patients there is a subpopulation of cancer cells expressing high CD44 and low or no CD24, that enables formation of a tumor *in vivo* limiting dilution assay^189^. CD44 has also been identified as a CSC marker in CRC^166–168^, pancreatic cancer^169^, ovarian cancer^170^, gastric cancer^171,172^, prostate cancer^157,173^, NSCLC^174^, OSCC^175^, and NPC^176^. The combination of CD133 and CD44 more specifically defines CSCs in CRC and HCC^168,177,178^. In fact, this combination has been used to define a subpopulation of HCC with high intrahepatic or lung metastatic capacity^304^. Several splicing variants of CD44 have been generated through alternative splicing in the membrane-proximal stem region. Exons 1-5 and 6-20 are spliced together and translated into the standard isoform, CD44s. Alternatively, exons 6-15 can be spliced to yield variant isoforms (labeled CD44v) along with the standard isoform^305^. CD44v8-10 have been identified as human gastric CSC markers that contribute to tumor initiation^306^. CD44v6+ has also been reported as a CSC marker for colon cancer and HCC^90,307^. CD44s, CD44v4, and CD44v9 at the invasive tumor front are associated with poor prognosis in gastric cancer patients and CD44s-expressing CSCs exhibit mesenchymal properties^308^. CD44s has also been suggested to contribute to mesenchymal properties and metastasis in breast cancer and CRC^309,310^.

#### EpCAM

EpCAM is a cell–cell adhesion molecule expressed in healthy epithelial cells. Increasing evidence has shown EpCAM to be a CSC marker for numerous cancers, such as breast cancer^189,190^, CRC^166^, HCC^188,191^, NPC^176^, and pancreatic cancer^192,193^.

#### CD90

CD90, also known as thymocyte differentiation antigen-1 (Thy-1), is a glycosylphosphatidylinositol (GPI)-anchored glycoprotein belonging to the immunoglobulin superfamily. CD90 and other markers, such as Oct4, Sox2, and ALDH1, are upregulated in enriched CSCs when tumor cells are cultured in tumorsphere-forming conditions. This finding suggests a vital role for CD90 as a marker for CSCs^181^. CD90 has been identified in CSCs of several cancers, such as murine breast cancer^179^, HCC^180^, gastric cancer^181^, esophageal squamous cell carcinoma (ESCC)^182^, lung cancer^183^, pancreatic cancer^184^, gliomas^185,186^, and insulinomas^187^. According to the study by Yamashita et al.^188^, EpCAM^+^ and CD90^+^ CSCs in HCC have distinct phenotypes and metastatic potential. Other studies have revealed that CD90^+^/CD44^+^ HCC cells are more aggressive and likely to metastasize to the lung. The combination of CD90^+^/CXCR4^+^ is more specific for defining circulating CSCs in HCC^180,311^.

#### Lgr5

Lgr5 is a transmembrane receptor belonging to the rhodopsin family of G protein-coupled receptors. Lgr5 has a pivotal role in normal embryonic development that was first known as a marker of intestinal stem cells^312^. Lgr5 is also highly expressed in various cancer tissues and enhances tumorigenesis, cancer cell mobility, and EMT in breast cancer cells by activating multiple pathways, such as Wnt/β-catenin and Notch signaling. Recent evidence also suggests that Lgr5 has a significant role in maintaining CSCs, making Lgr5 a CSC biomarker in numerous types of cancers, such as gastric cancer^194,195^, pancreatic cancer^196,197^, HCC^198,199^, CRC^200,201^, ovarian cancer^202^, cervical cancer^203^, and breast cancer^204^. de Sousa e Melo and colleagues^313^ reported that proliferative Lgr5^-^ CRC cells replenish Lgr5^+^ CSCs, resulting in rapid tumor recurrence upon treatment cessation. By analyzing the stemness properties of Lgr5^+^/CD44^+^/EpCAM^+^, Lgr5^+^/CD44^+^/EpCAM^−^, Lgr5^+^/CD44^-^/EpCAM^+^, Lgr5^−^/CD44^+^/EpCAM^+^, and Lgr5^-^/CD44^-^/EpCAM^−^ cells, Leng and colleagues^314^ concluded that Lgr5^+^ cells have greater potential for colony formation, self-renewal, differentiation, and tumorigenicity than Lgr5^−^ cells. The combination of Lgr5^+^/CD44^+^/EpCAM^+^ is a more specific marker of human CRC CSCs.

### Transcription factors

Numerous studies have revealed that multiple stemness-related transcription factors are abnormally expressed in cancers and associated with both CSCs and poor prognosis. Stem cells highly express approximately 25 transcription factors not found in healthy somatic cells.

#### Oct4

Oct4 is a transcription factor encoded by the *Pou5f1* gene. Oct4 belongs to the POU-homeodomain family and binds to an octamer motif, ATGCAAAT. Oct4 has a crucial role in maintaining pluripotency and self-renewal of both embryonic stem cells (ESCs) and CSCs^315–318^. Additionally, Oct4 induces tumorsphere formation, EMT, tumorigenesis, and resistance to chemo- or radio-therapy^238,319,320^. Oct4 is highly expressed in CSCs of various human cancers, such as glioma^205^, pancreatic cancer^206^, HCC^207^, breast cancer^208^, prostate cancer^209,210^, bladder cancer^211^, ovarian cancer^212^, and lung cancer^213^.

#### Sox2

Sox2 is one of the core transcription factors associated with pluripotency. Sox2 has a vital role in maintaining self-repair and proliferation of CSCs in various human cancers^321^. Sox2 also has oncogenic roles^322,323^. In lung cancer, Sox2 is highly linked with the ‘lineage-specific survival mechanism’ in lung cancer. Sox2, with or without mutated Lkb1, promotes mouse lung adenocarcinoma progression into squamous cell carcinoma (SCC) through pathologically mixed intermediates^324^. SOX2 expression characterizes CSCs in various cancers, including HNSCC^214^, medulloblastoma^215^, glioma^216^, breast cancer^217^, gastric cancer^218^, CRC^219^, lung cancer^220^, cervical cancer^221^, melanoma^222^, osteosarcoma^223^, ovarian cancer^224^, pancreatic cancer^225^, bladder cancer^226^, and skin cancer^227^.

#### Krüppel-like factor 4 (Klf4)

Klf4 is one of four crucial transcription factors involved in maintaining pluripotency in embryonic cells^325^. Klf4 is a bifunctional transcription factor that belongs to the Krüppel-like factor family. In the intestinal and gastric epithelium, Klf4 acts as a tumor suppressor^326^. Klf4 expression declines in leukemia^228^, anaplastic meningioma^229^, CRC^230^, gastric cancer^231^, NSCLC^232^, HCC^233^, bladder cancer^234^, and ESCC^235^. In melanoma and canine mammary tumors, Klf4 promotes tumorigenesis. In melanoma xenografts, Klf4 knockdown inhibits tumor growth *in vivo*^327^. In canine mammary tumors, highly overexpressed Klf4 is related to a more aggressive phenotype^328^. These findings indicate that Klf4 has a complex role in CSCs.

#### Nanog

Nanog has a crucial role in maintaining pluripotency^329^. Nanog functions in tandem with other regulators in CSCs, such as Sox2, Oct4, kinases, and miRNAs, to mediate the stemness phenotype through several signaling pathways, such as TGF-β, Wnt/β-catenin, JAK/STAT, Notch, and Hedgehog^330–332^. Overexpression of Nanog combined with Wnt1 leads to the initiation of breast tumors, but Nanog alone does not lead to tumorigenesis^333^. Nanog is often used as a stemness-associated reporter and is ubiquitously found in tumors, including GBM^236^, leukemia^237^, lung cancer^238^, breast cancer^239^, ESCC^240^, gastric cancer^241^, CRC^242^, ovarian cancer^243^, prostate cancer^244^, HCC^245^, HNSCC^246^, and renal cancer^247^.

#### c-Myc

The Myc gene family comprises three members (C-Myc, N-Myc, and L-Myc). These members exert an oncogenic role by regulating various cellular processes, such as the cell cycle, cellular survival, proliferation, and metabolic reprogramming^334–338^. c-MYC is expressed in CSCs of multiple cancers, including neuroblastoma^248^, lung cancer^249^, CRC^250^, and breast cancer^251^.

### Other markers in CSCs

In addition to cell surface molecules and transcription factors, studies have identified other CSC markers, including ALDH, Bmi-1, Nestin, Musashi-1, T-cell immunoglobulin mucin-3 (TIM-3), and CXCR4.

#### ALDH

ALDH is an enzyme that is involved in intracellular aldehyde detoxification and retinoic acid synthesis. ALDH is a single marker of CSCs in HNSCC and lung cancer^339,340^. ALDH^+^ cells exhibit signatures of both leukemia stem cells and hematopoietic stem cells in acute myeloid leukemia (AML), whereas ALDH^−^ cells mainly show progenitor cell signatures, indicating that ALDH^+^ AML originates from stem cells^341^. The ALDH family is composed of 19 members with ambiguous functions in cancer. Increasing evidence has shown that ALDH1A1 can be used as a CSC marker in a panel of cancers, including ovarian cancer^252,253^, lung cancer^254,255^, breast cancer^257^, cervical cancer^259^, and HCC^260^. ALDH1A3 is another CSC marker found in cancers of the breast^258^, lung^256^, and colon^261^. Moreover, ALDH1B1 has been referred to as a CSC marker in colon cancer^262^.

#### Bmi-1

Bmi-1 is a member of polycomb repressor complex I and is considered a proto-oncogene predominantly expressed in CSCs and essential for self-renewal and clonal expansion^342,343^. Bmi-1 overexpression leads to EMT and enhances cancer stemness in the NSCLC cell line, A549^344^. Activation of Bmi-1 has also been found in breast CSCs characterized by CD44^+^/CD24^−/low^/Lin^−^^345^. Targeting Bmi-1^+^ CSCs inhibits the growth and eliminates chemotherapy resistance in HNSCC^342^. The cancers characterized by Bmi-1 include gastric cancer^263^, HNSCC^5,264^, CRC^265^, ESCC^266–268^, hematopoietic neoplasms^269^, and glioma^270,271^.

#### Nestin

Nestin, an intermediate filament protein, was initially described as a neuronal stem cell or progenitor cell marker in 1990^346^. Nestin is often co-expressed with other stem cell markers, such as CD133, Oct3/4, and Sox-2 in various human solid tumors, including neurogenic tumors^272–276^, rhabdomyosarcoma^277^, osteosarcoma^278^, chondrosarcoma^160^, fibrosarcoma^160^, ovarian cancer^279–281^, OSCC^282^, prostate cancer^283,284^, gallbladder cancer^285^, lung cancer^286^, colon cancer^287^, breast cancer^288,289^, gastric cancer^290^ and pancreatic cancer^291^.

#### Musashi-1 (Msi1)

The RNA-binding protein, Msi1, is involved in post-transcriptional gene regulation by competing with eukaryotic translation initiation factor 4G (eIF4G). Kanemura et al.^347^ and Toda et al.^348^ reported the prognostic significance of Msi1 and MIB1 in human gliomas. Msi1 is considered a stem cell marker regulating homeostasis between self-renewal and differentiation^349^. Msi1 is expressed in CRC^292^, OSCC^293,294^, and neuronal cancer CSCs^295^.

#### Tim-3

Tim-3 signaling regulates immune responses by downregulating interferon production, thereby inducing T-cell exhaustion^350,351^. CD44^+^CD90^+^ CSC-like cells and T cells exhibit increased TIM-3 in SCLC^60^. TIM-3 is highly expressed on LSCs from most AML patients, but not those with acute promyelocytic leukemia, and is generally not expressed on normal hematopoietic stem cells^296^.

#### CXCR4

CXCR4 belongs to an important subfamily of chemokine receptors that consist of seven mutually parallel, tightly arranged transmembrane-spanning segments that are closely related to cancer progression and prognosis^352,353^. CXCR4 is a GPCR chemokine receptor regulating leukocyte trafficking, stem cell mobilization, and homing of stem cells^354,355^. CXCR4 keeps stem cells in niches by interacting with SDF-1^356^. The expression of CD44 and CD133 is associated with a high level of CXCR4 in prostate CSCs^357^. CD133^+^/CXCR4^+^ CSCs promote metastasis in CRC^358^. The same subpopulation has also been identified in pancreatic cancer, where CD133^+^/CXCR4^+^ CSCs have a high tendency to metastasize^164^. Moreover, CXCR4 is a CSC marker in glioma^297^ and prostate cancer^298^.

Novel biomarkers of CSCs include SIRYγ and OSMR in lung cancer and glioblastoma, respectively^6,359^. These new biomarkers help us identify specific CSC subpopulations in distinct cancers that express different phenotypic markers for CSCs with higher accuracy. However, current biomarkers are not specific enough, so comprehensive studies are needed to identify more accurate biomarkers, either individually or in combination, for identifying CSCs.

## Mechanisms of therapeutic resistance in CSCs

Multiple stemness-related signaling pathways initially found in normal stem cells have been validated in CSCs, including the Hedgehog, Wnt, Notch, and NF-κB pathways, which also have crucial roles in therapeutic resistance and have been extensively reviewed elsewhere^360,361^. Mechanisms of therapeutic resistance are complex and involve activation of survival signaling, evasion of apoptosis, high activities of acetaldehyde dehydrogenase, cell dormancy, disrupted cell differentiation, abnormal DNA damage/repair, altered epigenetic modification, immune suppression, inhibition of ROS, and hypoxia. For example, ROS scavengers in CSCs partially block the increase in ROS and protect CSCs from permanent damage to DNA, RNA, and other biomacromolecules^362^. The earliest finding in this regard was that ATP-binding cassette efflux transporters (ABC transporters) are the membrane proteins in bacteria. ABC transporters were subsequently shown to have an essential role in resistance to chemotherapy by efflux of drugs in humans^363,364^. ABC transporters (ABCB1, ABCC2, and ABCG2) overexpressed in CSCs is regarded as a CSC biomarker and the predictor of chemotherapeutic resistance^365,366^. Vasculogenic mimicry (VM), referring to the replacement of endothelial cells by tumor cells and the creation of a vessel with a lumen, can be induced by VM-related molecules secreted by CSCs, such as VE-cadherin protein, which ultimately confer resistance to antiangiogenic therapies and other anti-cancer therapies^367–370^. In this section, we mainly review the mechanisms underlying resistance to radio- and chemo-therapy.

### Mechanisms of radiotherapeutic resistance in CSCs

**Figure 3:**
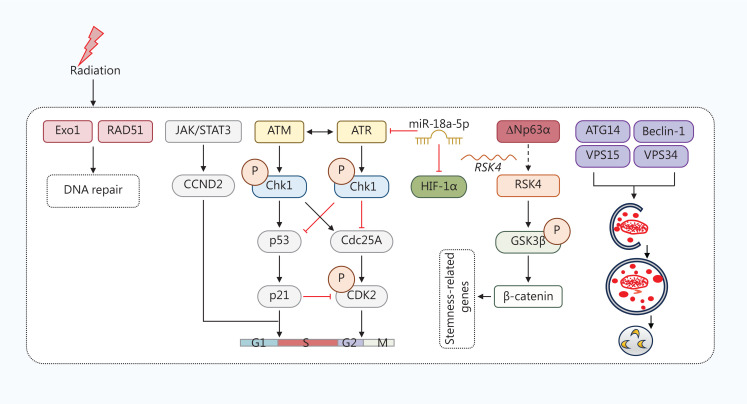
The mechanisms underlying radio-resistance in CSCs. Upon radiation, multiple signaling pathways are triggered to induce resistance and maintain stemness in CSCs. First, radiation induces the expression of Exo1 and RAD51, which are involved in DNA repair. The JAK/STAT3 pathway is triggered by radiation, then activates the following CCND2 signaling and subsequent G1-to-S transition. Radiation also alters the cell cycle through ATM/ATR-associated signaling. In CSCs, miR-18a-5p overexpression degrades ATR and HIF-1α, thereby overcoming resistance to radiation. Additionally, ΔNp63α promotes the transcription of ribosome S6 protein kinase 4 (RSK4). Then, RSK4 phosphorylates GSK-3β at Ser9, which leads to the transcription of β-catenin-mediated stemness-related genes. Moreover, autophagy-related proteins are upregulated in some CSCs, where CSCs are involved in radio-resistance.

A multicenter study has shown that CD44, a CSC marker in laryngeal cancer, predicts an increase in the CSC population and acquires radio-resistance and recurrence after radiotherapy in patients at an early stage^371^. In HER2-expressing breast CSCs (HER2^+^/CD44^+^/CD24^−/low^), a significant reduction in cell sensitivity to irradiation (IR)-induced apoptosis and increased clonogenic survival were observed when compared to wild-type MCF7 cells^372^. Similarly, in lung cancer cell lines, exposure to a single 4 Gy of radiation enriched CD133^+^ CSCs with increased DNA repair. Homologous recombination (HR)-associated proteins [exonuclease 1 (Exo1) and RAD51] have been identified as key regulators of radio-resistance in CSCs (**Figure 3**)^373^. Ataxia–telangiectasia mutated (ATM) and ATM-RAD3-related (ATR) are well known as upstream proteins of checkpoints that respond to different types of DNA damage. ATM, ATR, and the downstream checkpoint kinases (Chk1 and Chk2) mediate radio-resistance. Rapid induction of Chk1 is associated with enhanced G2/M cell cycle checkpoint activation in gastric CSCs. Chk1 inhibition sensitizes CSCs to IR^374–376^. Chk1 and Chk2 facilitate the DNA damage response by initiating cell cycle checkpoint control and activating the corresponding DNA repair pathways. Enhanced ATM signaling that contributes to the specific DNA repair phenotype is also observed in glioma CSCs with radio-resistance^377^. Chk1 knockdown in CD133^+^/CD44^+^ prostatic CSCs abrogates radiation-induced G2/M arrest, inhibits DNA damage repair, and promotes premature mitosis, leading to increased apoptosis^378^. Furthermore, inhibition of ATM overcomes radio-resistance in breast CSCs^379^. A mechanistic study reported that breast CSCs have a 7-fold higher ATM phosphorylation in response to 2 Gy radiation over non-CSCs^379^. Cyclin D2 (CCND2), a member of the cyclin protein family, has an important role in promoting colorectal CSCs to survive after radiation by activating cell cycle progression, DNA replication, and DNA repair. Targeting the JAK2/STAT3/CCND2 pathway overcomes radio-resistance by resisting radiation-induced apoptosis^380^. Inhibition of autophagy by silencing beclin1 and autophagy-related 5 (ATG5) or bafilomycin A1 increases the sensitivity of CD133^+^ gastric CSCs to radiation^381^. HIFs mainly regulate CSCs from gastric cancer and CRC in the hypoxic microenvironment, which causes chemo- and radio-resistance^382,383^. Additionally, miR-99a modulates breast CSC self-renewal by suppressing HIF-1α and mTOR signaling^384^, while miR-18a-5p overexpression increases the radiosensitivity of CD133^+^ lung CSCs by downregulating HIF-1α and ATM *in vitro* and *in vivo*^385^. Furthermore, the ΔNp63α/ribosome S6 protein kinase 4 (RSK4)/glycogen synthase kinase 3β (GSK-3β) axis contributes to CSC properties and radio-resistance in ESCC, suggesting that RSK4 is a promising therapeutic target^386^. The roles of different signaling pathways associated with CSCs (STAT3, PI3K/Akt/mTOR, ERK, VEGF, Notch, and Wnt/β-catenin pathways) in radio-resistance have been summarized by Chang et al.^387^.

### Mechanisms of chemoresistance in CSCs

**Figure 4:**
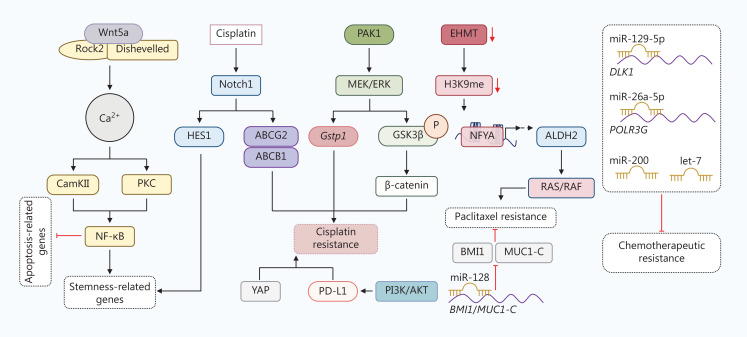
The mechanisms underlying chemoresistance in lung CSCs. Extracellular Wnt5a initializes the Ca^2+^ pathway by interacting with Rho-kinase 2 (Rock2) and dishevelled on the membrane. Ca^2+^ then activates CamKII and protein kinase C (PKC), which induce nuclear factor kappa B (NF-κB)-mediated transcription of stemness-related genes and inhibit the transcription of apoptosis-related genes. Upon cisplatin treatment, Notch1 is upregulated in CSCs. Notch1 promotes stemness in a hairy and enhancer of split (HES1)-dependent manner and triggers cisplatin resistance by increasing ATP-binding cassette transporter G2 (ABCG2) and ABCB1. p21-activated kinase 1 (PAK1) activates the MEK/ERK signaling pathway in CSCs, followed by the elevated transcription of Gstp1 or activation of β-catenin signaling regulated by phosphorylation of GSK3β at Ser9, which leads to cisplatin resistance. Moreover, Yes1-associated transcriptional regulator (YAP1) and PI3K/AKT-induced PD-L1 are reported to cause cisplatin resistance. Euchromatic histone lysine methyltransferase (EHMT), which catalyzes the methylation of H3K9, is reduced in CSCs. Nuclear transcription factor Y subunit A (NFYA) is then recruited to DNA and initializes transcription of ALDH2, which results in paclitaxel resistance through RAS/RAF signaling. Several microRNAs (miRNAs), including miR-128, miR-129-5p, miR-26a-5p, miR-200, and let-7, impede chemoresistance in CSCs.

During the process of treatment, chemotherapeutic agents often induce CSCs enrichment^366^. For example, doxorubicin treatment elevates the proportion of EpCAM^+^/CD133^+^ cells in HCC Huh7^388^. CSCs expressing Sox2 are resistant to tamoxifen, an antagonist of the estrogen receptor, in breast cancer through activation of the Wnt signaling pathway^389^. The intrinsic and induced enrichment of CSCs contributes to chemotherapeutic resistance. The mechanisms of chemoresistance in CSCs vary by cancer type. Here, we mainly focus on chemoresistant mechanisms in CSCs from lung cancer (**Figure 4**).

Mesenchymal NSCLC cells are resistant to epithelial growth factor receptor (EGFR), tyrosine kinase inhibitors (TKIs), erlotinib, and cisplatin compared to parental cells^390^. Platinum-based chemotherapy, paclitaxel, and etoposide are commonly used as first-line chemotherapy for lung cancer. A study showed that the development of cisplatin and etoposide resistance is associated with increased expression of the stem marker, CD133, *in vitro*^391^. ALDH1^+^ cells display greater resistance to the chemotherapeutic drugs, including cisplatin, gemcitabine, doxorubicin, daunorubicin, vinorelbine, and docetaxel, than ALDH1^−^ cells^392^. The CSC biomarker, ALDH2, has been shown to contribute to paclitaxel resistance through the RAS/RAF pathway in lung cancer. The ALDH2 inhibitors, daidzin (DZN) and disulfiram (DSF), reverse paclitaxel resistance in xenograft models by promoting cell apoptosis and blocking the RAS/RAF pathway^393^. Cisplatin elevates the proportion of CD133^+^ cells by activating Notch1 signaling and upregulating ABCG2 and ABCB1 expression, which lead to cross-resistance to doxorubicin and paclitaxel^394^. Hashida et al.^395^ reported that established afatinib-resistant cells exhibit characteristics of EMT and stemness. Afatinib-resistant cells also exhibit amplification of MET genes, express high levels of ALDH1A1 and ABCB1, and are resistant to chemotherapeutic agents. Upregulated ABCG2 also facilitates resistance by effluxing gefitinib in NSCLC cells^396^. Several signaling pathways modulate chemoresistance in CSCs, such as YAP, Erk, and Notch.

Knockdown of YAP sensitizes A549 tumorspheres to cisplatin in NSCLC cells^397^. p21-activated kinase 1 (PAK1) confers cisplatin resistance in NSCLC cells. PAK1 activates MEK/ERK signaling, which promotes phosphorylation of GSK3β at Ser9 and subsequent β-catenin-mediated stemness^398^. Zhang and colleagues^399^ have detected a CD166^+^CD49f^high^CD104^−^Lin^−^ subpopulation with CSC properties from patient-derived sphere-forming assays. This subpopulation exhibits a high level of Notch1 and its ligand, delta-like canonical Notch ligand 4 (DLL4), which maintains self-renewal and platinum resistance through the Notch1 intracellular domain (NICD1)/hairy and enhancer of split (HES1)/STAT3 axis and survival regulators, respectively^399^. MEK/ERK signaling confers cisplatin resistance by transcriptionally inducing glutathione S-transferase Pi (*Gstp1*) in murine lung cancer cell LLC-derived CSCs^400^. Furthermore, activation of Wnt/β-catenin signaling is a mechanism resulting in cisplatin resistance. Silencing β-catenin sensitizes A549 cells to cisplatin by blocking Bcl-xl^401^. The non-canonical Wnt5a/PKC signaling pathway promotes stemness and cisplatin resistance in cisplatin-resistant A549 cells^402^. Recent studies have shown that upregulation of PD-L1 by the PI3K/AKT pathway is the main cause of cisplatin resistance in lung cancer cells^403^. The Wnt/β-catenin and Shh signaling pathways are commonly hyperactivated in CSCs. Furthermore, PD-L1 overexpression in CSCs contributes to immune evasion. Therefore, we speculate that Wnt/β-catenin signaling, the Shh signaling pathway, and PD-L1 might also have a role in cisplatin resistance in CSCs. Moreover, downregulated miR-129-5p, miR-26a-5p, miR-128, miR-200, and let-7 family miRNAs contribute to stemness and chemoresistance in stem-like NSCLC cells^390,404–406^. *In silico* prediction and *in vitro* experiments have identified the Notch signaling receptor, delta-like 1 homolog (DLK1), and RNA polymerase III subunit G (POLR3G) as targets of miR-129-5p and miR-26a-5p, respectively^405,406^. miR-128 suppresses BMI1 and MUC1-C expression, thereby weakening CSC-related traits in paclitaxel-resistant lung CSCs^404^. BRM270 extracted from herbal plants has been reported to induce miR-128 expression in chemoresistant CSCs, which overcomes paclitaxel resistance^407^.

## Prospects of targeting CSCs

Because CSCs are the major cell population giving rise to therapeutic resistance, many studies have explored effective therapeutic strategies for targeting CSCs. Inhibitors targeting stem-associated signaling pathways (Shh, Wnt/β-catenin, and Hippo) have been evaluated preclinically and clinically, and summarized elsewhere^408^. Surface molecules are not only critical biomarkers for CSC isolation but also potential targets for treatment^409^. Additionally, the interplay between CSCs, the TME, and metabolism has given us promising therapeutic targets. In this section we mainly summarize the current treatments targeting the TME and CSC metabolism.

### Targeting the TME of CSCs

Effective treatment for immunosuppressive tumor phenotypes can be challenging due to poor T-cell priming or immunologic ignorance. Single agents blocking PD-1 or PD-L1 performed poorly at converting “cold” tumors to “hot” tumors^410^. A PD1-based CSC vaccine has been shown to inhibit tumor growth in an animal colon cancer model^411^. Combining immune checkpoint blockade and CSC targeting therapies could be a promising treatment for immunosuppressive tumor phenotypes.

Various immune-based therapeutic strategies have been investigated to target CSCs^412^ (**Table 2**). T cell-based therapies, such as adoptive cell transfer therapy (ACT), have proven effective at fighting cancer. This form of personalized cancer treatment involves the administration of *ex vivo* expanded autologous tumor-infiltrating lymphocytes to cancer patients. CAR-T cells are patient-derived T cells engineered to express antibodies against desired cell surfaces. Human CAR-T cells targeting EpCAM have the potential to eradicate established tumor xenografts without causing toxicity in mouse models^413,414^. CAR-T cells expressing EpCAM accumulate in prostate tumors and eradicate CSCs in PC3M and PC3^413^. The adoptive transfer of γδ and CD8^+^ T cells also upregulates MHC class I and CD54/ICAM-1 on CSCs and activates antigen-specific T-cell killing^415^.

**Table 2:** Ongoing clinical trials of investigational CSC-directed immunotherapeutic approaches

Agent	Disease	Sample size	Phase	NCT number	Current status
CD19 CAR-T	Relapsed/refractory non-Hodgkin lymphoma (NHL)	82	II	NCT04089215	Recruiting
CD123 CAR-T	Relapsed/refractory acute myelogenous leukemia (AML)	40	I/II	NCT04272125	Recruiting
CD133 CAR-T	Relapsed/refractory advanced malignancies	20	I/II	NCT02541370	Completed
CD22 CAR-T	B cell malignancies	20	I/II	NCT03262298	Recruiting
	Childhood leukemia	100	II	NCT04340167	Recruiting
CD33 CAR-T	Relapsed/refractory AML	25	I/II	NCT04835519	Recruiting
CD38 CAR-T	Relapsed B-cell acute lymphocytic leukemia (ALL) after CD19 CAR-T adoptive cellular immunotherapy	80	I/II	NCT03754764	Unknown
BCMA-CART	Relapsed/refractory multiple myeloma (MM)	150	I	NCT04394650	Active, not recruiting
Mesothelin CAR-T	Advanced refractory solid tumors	12	I	NCT04981691	Recruiting
	Mesothelioma	30	I	NCT04577326	Recruiting
LeY CAR-T	Advanced solid tumors	20	I	NCT03851146	Completed
CD30 CAR-T	Relapsed/refractory Hodgkin lymphoma	97	II	NCT04268706	Active, not recruiting
	CD30-expressing lymphomas	26	I	NCT03049449	Completed
	Relapsed/refractory Hodgkin and T-cell lymphoma	30	I/II	NCT04653649	Recruiting
CD70 CAR-T	CD70-positive malignant hematologic diseases	108	I	NCT04662294	Recruiting
CD71 CAR-T	CD70-positive advanced/metastatic solid tumors	48	I	NCT05468190	Recruiting
CD171 CAR-T	Neuroblastoma/ganglioneuroblastoma	65	I	NCT02311621	Active, not recruiting
CXCR4 CAR-T	Refractory/relapsed MM		I	NCT04727008	not recruiting
AMG 119 (anti-DLL3) CAR-T	Small-cell lung cancer (SCLC)	6	I	NCT03392064	Suspended
c-Met/PD-L1 CAR-T	Hepatocellular carcinoma (HCC)	50	I	NCT03672305	Unknown
GD2 CAR-T	Relapsed/refractory neuroblastoma or other GD2-positive solid tumors	42	I/II	NCT03373097	Recruiting
	Diffuse intrinsic pontine gliomas (DIPG) and spinal diffuse midline glioma (DMG)	54	I	NCT04196413	Recruiting
	Lung cancer	24	I	NCT05620342	Not yet recruiting
GPC3 CAR-T	Advanced HCC	38	I	NCT05003895	Recruiting
GPC4 CAR-T	Pediatric HCC	10	I	NCT02932956	Active, not recruiting
MOv19-BBz CAR-T	aFR expressing recurrent high grade serous ovarian, fallopian tube, or primary peritoneal cancer	18	I	NCT03585764	Recruiting
P-MUC1C-ALLO1 CAR-T	Advanced or metastatic solid tumors	100	I	NCT05239143	Recruiting
IL13Ralpha2 CAR T	Leptomeningeal glioblastoma, ependymoma, or medulloblastoma	30	I	NCT04661384	Recruiting
EpCAM CAR-T	Advanced gastric cancer with peritoneal metastasis	40	I	NCT03563326	Recruiting
	EpCAM-positive cancer	60	I/II	NCT03013712	Completed
BCMA CAR-T	BCMA-positive relapsed/refractory MM	28	I	NCT03338972	Completed
NK cells	Advanced lung adenocarcinoma (LUAD) with an EGFR mutation	10	I	NCT03662477	Unknown
	Recurrent glioblastoma multiform patients	5	I	NCT05108012	Recruiting
	HCC	18	I	NCT02399735	Unknown
Cytokine-induced killer cell (CIK)	After resection of liver cancer	200	III	NCT00769106	Completed
	SCLC	60	II	NCT01592422	Completed
	Urinary bladder neoplasms	1500	II	NCT02489890	Active, not recruiting
DC vaccine ALDH	Colorectal cancer (CRC)	40	I/II	NCT02176746	Completed
	Ovarian cancer	40	I/II	NCT02178670	Completed
	Breast cancer	40	I/II	NCT02063893	Completed
	Lung cancer	40	I/II	NCT02084823	Completed
	Nasopharyngeal carcinoma (NPC)	40	I/II	NCT02115958	Completed
	Pancreatic cancer	40	I/II	NCT02074046	Completed
	Liver cancer	40	I/II	NCT02089919	Completed
Multiantigen DNA plasmid-based vaccine (CD105, Yb-1, SOX2, CDH3, and MDM2)	HER2-negative breast cancer	42	I	NCT02157051	Active, not recruiting
DCs pulsed with lysate from allogeneic glioblastoma stem-like cell line	Newly diagnosed or recurrent glioblastoma	39	I	NCT02010606	Completed
CSC vaccine	Pancreatic cancer	40	I/II	NCT02074046	Completed
	NPC	40	I/II	NCT02115958	Completed
	Lung cancer	40	I/II	NCT02084823	Completed
	HCC	40	I/II	NCT02089919	Completed
	CRC	40	I/II	NCT02176746	Completed
	Ovarian cancer	40	I/II	NCT02178670	Completed
	Breast cancer	40	I/II	NCT02063893	Completed
OH2 oncolytic viral therapy	Solid tumors	300	I/II	NCT03866525	Recruiting
	Pancreatic cancer	25	I/II	NCT04637698	Recruiting
	Advanced bladder carcinoma	45	II	NCT05248789	Recruiting
	Central nervous system tumors	28	I/II	NCT05235074	Recruiting
Wild-type reovirus	Metastatic melanoma	23	II	NCT00651157	Completed
MV-NIS	Recurrent medulloblastoma or recurrent atypical teratoid rhabdoid tumor (ATRT)	46	I	NCT02962167	Recruiting
	Bladder cancer who are undergoing radical cystectomy	16	I	NCT03171493	Recruiting
Oncolytic adenovirus Ad5-DNX-2401	Recurrent high-grade glioma	36	I	NCT03896568	Recruiting
Oncolytic adenovirus ONCOS-102 and pembrolizumab	Advanced or unresectable melanoma progressing after PD1 blockade	21	I	NCT03003676	Completed
Oncolytic adenovirus VCN-01	Refractory retinoblastoma	13	I	NCT03284268	Recruiting
Oncolytic adenovirus ColoAd1	CRC/non-small cell lung cancer (NSCLC)/urothelial cell cancer (UCC)/renal cell carcinoma (RCC)	17	I	NCT02053220	Completed
Oncolytic adenovirus OBP-301	Advanced esophageal cancer and are not candidates for surgery	12	I	NCT04391049	Recruiting
HSV-1716	Malignant pleural mesothelioma	12	I/II	NCT01721018	Completed
	Non-central nervous system (non-CNS) solid tumors	18	I	NCT00931931	Completed
HSV-M032	Recurrent malignant glioma	24	I	NCT02062827	Active, not recruiting
Hu5F9-G4	Solid tumor	88	I	NCT02216409	Completed
	Hematologic malignancies	20	I	NCT02678338	Completed
SRF231	Advanced solid and hematologic cancers	148	I	NCT03512340	Completed
TTI-621	Hematologic malignancies and selected solid tumors	250	I	NCT02663518	Active, not recruiting
TTI-621 with or without rituximab	Advanced hematologic malignancies, including lymphoma, leukemia, and MM	476	I	NCT03530683	Recruiting
ALX148	Advanced solid and hematologic cancers	60	I	NCT02367196	Completed
ALX149	Advanced solid tumors and lymphoma	174	I	NCT03013218	Active, not recruiting
Reparixin	Metastatic breast cancer	33	I	NCT02001974	Completed
	Metastatic triple-negative breast cancer (TNBC)	194	II	NCT02370238	Completed
KHK2823	AML	39	I	NCT02181699	Terminated
Talacotuzumab	AML	30	I	NCT01632852	Completed
	AML	326	II/III	NCT02472145	Completed
Flotetuzumab	AML	246	I/II	NCT02152956	Terminated
XmAb14045	CD123-expressing hematologic malignancies	120	I	NCT02730312	Completed
JNJ-63709178	Relapsed or refractory AML	62	I	NCT02715011	Completed
MT110	Solid tumors	65	I	NCT00635596	Completed
AMG 757	Neuroendocrine prostate cancer	60	I	NCT04702737	Recruiting
	Extensive stage SCLC	340	I	NCT05361395	Recruiting
CD133+ stem cell transplantation, busulfan, and melphalan	Children with solid tumors and lymphomas	26	Not applicable	NCT00152126	Completed
CD133+ stem cell transplantation and portal vein embolization	Colorectal liver metastases	4	II	NCT03803241	Completed
IFN-β therapy	Metastatic cutaneous melanoma or ocular melanoma	21	II	NCT00085306	Completed
SBRT with anti-PD1 and anti-IL-8	Multiple metastases in advanced solid tumors	50	I	NCT04572451	Recruiting
Nivolumab with anti-IL-8	NSCLC or HCC	50	II	NCT04123379	Recruiting
MCLA-158	Advanced solid tumors	120	I	NCT03526835	Recruiting

Oncolytic virus (OV) is another immunotherapy with low toxicity that targets and destroys tumor cells through cytopathic effects in a direct and indirect fashion^416^. The OV, GLV-1h68, can kill stem cell-like cancer cells (higher ALDH1 activity) in breast cancer. In cell culture, GLV-1h68 replicates in and kills breast CSCs^417^.

In addition to T cells, other immune cells, such as NK cells, B lymphomas, macrophages, and neutrophils, have roles in shaping the TME in cooperation with CSCs, as mentioned above. DCs were treated with CSC lysates and tumor-related antigens *ex vivo* to generate a DC-based vaccine, which was then injected back into cancer patients^418^. CSC lysate-pulsed DCs induced IFN-γ and IL-4 secretion in vaccinated mice with malignant melanoma, inhibiting tumor growth and prolonging survival in immunized mice^419^. Secretion of INF-γ and IL-2 induced by pancreatic CSC lysate-loaded DC vaccination promotes the function of lymphocytes in pancreatic cancer cells^420^. Similarly, DCs charged with Nanog peptides enhance the anti-tumor activity of T cells against CSCs in ovarian cancer^421^. DCs loaded with ALDH^high^ SCC7 have also been reported to reduce recurrence and prolong survival in a murine HNSCC model^422^.

Moreover, the adoptive transfer of NK cells causes an improvement in MICA/B, Fas, and DR5 as NK cell-activating ligands on CSCs^423^. Anti-CD133 CAR-engineered NK-92 cells kill CD133^+^ ovarian CSCs *in vitro* and *in vivo*. Cisplatin treatment followed by anti-CD133 CAR-engineered NK-92 cells significantly augment the anti-tumor effect in a murine ovarian model^424^. Although previous evidence has suggested the essential role of crosstalk between CSCs and TAMs or neutrophils, specific strategies that target the interaction remain unknown due to the ambiguous mechanisms of the interplay in individuals.

Signaling pathways have been reported in immune cells and CSCs, and are also promising targets, such as STAT3 and PI3K. Multiple STAT3 inhibitors have been developed and processed for clinical trials^425^. Notably, the first-in-class antisense oligonucleotide (ASO) targeting STAT3 AZD9150 has chemical stability and anti-tumor activity in several cancers. The efficacy of AZD9150 in various cancers is still ongoing or pending^426^. The efficacy of PI3K inhibitors, including PX-866 (IND205), alpelisib (NCT02437318), PQR309 (PQR309), and pictilisib (GDC-0941), has been tested in several clinical trials (**Table 2**). These PI3K inhibitors show considerable efficacy in some settings, especially in combination with inhibitors of other pathways, such as MEK, and require further investigation. CAFs are also a potential target for tumor treatment. Chen et al.^427^ have reported a cancer cell-targeted nanoliposome system that specifically targets and delivers Navitoclax (Nav) to CAFs.

### Targeting metabolism in CSCs

As mentioned above, heterogeneous metabolic patterns have been reported in CSCs of different types of tumors. Glycolysis is enhanced, which makes glycolysis a promising target in some CSCs. Targets of glycolysis include rate-limiting enzymes, transporters, and other complex regulators^428–430^. GLUT1, a glucose transporter, has an important role in the maintenance of pancreatic, ovarian, and GBM CSCs^431^. WZB117, a specific GLUT1 inhibitor, successfully inhibits the self-renewal and tumor-initiating capacity of the CSCs *in vitro*^432^. Silibinin, another GLUT1 inhibitor, causes the dual blockade of EMT and stemness of bladder CSCs *via* inactivation of β-catenin/ZEB1 signaling *in vitro*^433^. Phase I-II clinical trials have assessed the toxicity and efficacy of glucose transport inhibitors, such as WZB117, fisetin, phloretin, and silybin/silibinin, for advanced HCC and prostate cancer; however, these therapies have limitations due to side effects, such as hyperbilirubinemia and elevation of alanine aminotransferase (ALT)^434,435^. Blocking OXPHOS therapeutically suppresses CSC growth, including sphere and tumor formation potential^122,436,437^. Atovaquone, an U.S. FDA-approved anti-malarial drug and a selective OXPHOS inhibitor, has therapeutic efficacy against MCF7 breast cancer cells by targeting CoQ10-dependent mitochondrial complex III. Mitochondrial respiration is damaged, causing glycolysis to increase as compensation^438^. Mitochondrially-targeting antibiotics, including salinomycin, erythromycin, tetracyclines, and glycylcyclines, have been U.S. FDA-approved to reduce stemness characteristics in CSCs^437,439–441^. Metformin, an inhibitor of mitochondrial complex I, has been studied extensively for its potential to target CSCs. Metformin inhibits the self-renewal of CSCs in breast cancer by suppressing estrogen receptor-mediated Oct4 expression *in vitro*^442^. A recent study has suggested that targeting glutamine metabolism enhances the radiosensitization of prostate cancer cells by increasing DNA damage, shifting redox balance, and retarding CSC properties. Metformin also shows the capacity to inhibit both glutamine metabolism and autophagy in tumor cells^443^. Another approach to reversing the resistance of CSCs with an intermediate glycolytic/OXPHOS phenotype is the administration of menadione, an ROS inducer^122^.

Targeting CSCs through the inhibition of glutaminolysis, which is the process of converting glutamine-to-glutamate *via* the enzyme, glutaminase (GLS), is a promising metabolic interference strategy^431^. GLS1 is associated with different types of cancer, and the GLS1 inhibitor, BPTES, combined with the phosphodiesterase-5 inhibitor, zaprinast, increases the sensitivity of pancreatic CSCs to radiotherapy and promotes apoptosis by increasing the level of intracellular ROS^431^. The first glutaminase inhibitor, DON, was isolated from Peruvian soil and induces apoptosis of breast CSCs^444^. Additionally, nuclear factor-erythroid 2-related factor 2 (Nrf2), a redox-related transcription factor that regulates antioxidant enzymes for maintaining cellular redox status, has been linked to the regulation of CSCs^445–448^. In particular, the natural compound, honokiol, which is isolated from the wood of Cupressaceae trees, impedes the self-renewal, migration, and colony-forming ability of CSCs by inhibiting Nrf2 expression^449^. Similarly, the chestnut leaf inhibits sphere cell development and increases the chemosensitivity of breast CSCs to paclitaxel through inhibition of Nrf2 activity *in vitro*^450^.

FASN has a critical role in the production of endogenous fatty acids. Cerulenin is a natural antifungal antibiotic that has potent inhibitory properties against FASN^451^. Cerulenin curbs the self-renewal of gastric CSCs by suppressing adipogenesis^452^. Curcumin, in contrast, downregulates SCD1 and inhibits the self-renewal of breast CSCs^453^. Additionally, the SCD1 inhibitor, CAY10566, and the Δ6 desaturase inhibitor, SC-26196, inhibit the stemness of ovarian CSCs^454^. The vulnerability of cancer cells, particularly CSCs, to ferroptosis drives much effort to investigate the potential of ferroptosis as an anti-cancer strategy, although this vulnerability varies by cancer type. This finding has led to the investigation of many pathways, such as lipid metabolism, iron metabolism, and Nrf signaling, that regulate ferroptosis. Agents targeting these pathways have the potential to enhance the sensitivity of CSCs to ferroptosis. The combination of ferroptosis inducers with current treatments has been studied and reviewed by Lei et al.^142^ and Elgendy et al.^455^. The use of ferroptosis inducers requires careful investigation before clinical application because ferroptosis promotes tumor growth^135^.

Although there is still much to learn about metabolic patterns in CSCs, it is likely that targeting metabolism will be an effective strategy to treat cancer. Recent research suggests that drugs designed to target resistance regulatory pathways or abnormal proteins in CSCs could improve therapeutic outcomes. In addition to these therapies, the differentiation of tumor cells may be a promising approach. One such method is the use of all-trans retinoic acid (ATRA), which downregulates the stem cell markers, Oct4, Sox2, Nestin, and CD44, in HNSCC CSCs^456^. ATRA also enhances the chemosensitivity of HNSCC CSCs to cisplatin, suppresses the proliferation of CSCs *in vitro* and *in vivo*, and inhibits the Wnt/β-catenin pathway, resulting in a decrease in stemness^456^.

## Conclusions

Numerous attempts have been made to develop biomaterial-based platforms to enrich and study CSCs with the goal of targeting CSCs^457^. The potential of translating CSC biological research into clinical practice is quite promising. First, some biomarkers can distinguish CSCs from normal adult stem cells and cancer cells, but there is no definitive way to differentiate one from the other. Therefore, CSCs should be identified not only by the molecular and cellular biological features of normal stem cells but also by tumor-specific biomarkers with higher accuracy. Additionally, growing evidence indicates that CSCs have distinctive metabolic features in individual cancers, which suggests altered metabolism as a potential target. Some old drugs used for metabolic disorders, such as diabetes and natural compounds, have proven to be effective at targeting CSCs in preclinical trials. However, our understanding of the metabolic landscape in different types of cancers remains unclear. The metabolic patterns of CSCs, differentiated cancer cells, and stromal cells in the TME are not fully understood, which necessitates further investigation before clinical targeting of metabolism can be translated into practice. Moreover, all targeting strategies should take every compartment, including quiescent and proliferating CSCs, as well as the stromal cells and the TME matrix, into account. A combination of immunotherapies and other therapies against CSCs may improve the prognosis of patients and be widely available for clinical use. Precise drug delivery to CSCs enhances therapeutic accuracy and efficacy while minimizing damage to normal cells, which might be achieved through findings in chemistry and materials science, such as nanotechnology^458^.
